# Comparison of enteroendocrine cells and pancreatic β-cells using gene expression profiling and insulin gene methylation

**DOI:** 10.1371/journal.pone.0206401

**Published:** 2018-10-31

**Authors:** Gyeong Ryul Ryu, Esder Lee, Jong Jin Kim, Sung-Dae Moon, Seung-Hyun Ko, Yu-Bae Ahn, Ki-Ho Song

**Affiliations:** Division of Endocrinology & Metabolism, Department of Internal Medicine, College of Medicine, The Catholic University of Korea, Seoul, Korea; International University of Health and Welfare School of Medicine, JAPAN

## Abstract

Various subtypes of enteroendocrine cells (EECs) are present in the gut epithelium. EECs and pancreatic β-cells share similar pathways of differentiation during embryonic development and after birth. In this study, similarities between EECs and β-cells were evaluated in detail. To obtain specific subtypes of EECs, cell sorting by flow cytometry was conducted from STC-1 cells (a heterogenous EEC line), and each single cell was cultured and passaged. Five EEC subtypes were established according to hormone expression, measured by quantitative RT-PCR and immunostaining: L, K, I, G and S cells expressing glucagon-like peptide-1, glucose-dependent insulinotropic polypeptide, cholecystokinin, gastrin and secretin, respectively. Each EEC subtype was found to express not only the corresponding gut hormone but also other gut hormones. Global microarray gene expression profiles revealed a higher similarity between each EEC subtype and MIN6 cells (a β-cell line) than between C2C12 cells (a myoblast cell line) and MIN6 cells, and all EEC subtypes were highly similar to each other. Genes for insulin secretion-related proteins were mostly enriched in EECs. However, gene expression of transcription factors crucial in mature β-cells, such as PDX1, MAFA and NKX6.1, were remarkably low in all EEC subtypes. Each EEC subtype showed variable methylation in three cytosine-guanosine dinucleotide sites in the insulin gene (*Ins2*) promoter, which were fully unmethylated in MIN6 cells. In conclusion, our data confirm that five EEC subtypes are closely related to β-cells, suggesting a potential target for cell-based therapy in type 1 diabetes.

## Introduction

Pancreatic β-cell replacement by pancreas or islet transplantation is the only curative treatment for type 1 diabetes mellitus; however, the limited number of pancreas donors is a major obstacle [1]. To overcome this problem, many researchers have tried to expand existing β-cells and to convert other cells to β-cells [2].

Enteroendocrine cells (EECs) represent the largest population of endocrine cells in the body. Many EEC subtypes have been described, each characterized by secretion of a distinct hormone. These include L cells producing glucagon-like peptide-1 (GLP-1), K cells producing glucose-dependent insulinotropic polypeptide (GIP), I cells producing cholecystokinin (CCK), G cells producing gastrin, and S cells producing secretin [3]. However, recent studies have revealed that a specific EEC subtype expresses more than one hormone, indicating a greater overlap between EEC subtypes than had been thought originally [4–6]. EECs are derived from the endoderm, and after birth, arise continually from stem cells in the crypts of the intestine. Interestingly, EECs and β-cells share expression of specific transcription factors during the course of differentiation in embryonic development and after birth. For example, Notch signaling and its associated transcription factors, such as MATH1, NGN3 and NEUROD1/BETA2, play critical roles in determining endocrine cell fate in both the gut and pancreas. Other transcription factors, including PDX1, PAX4, PAX6, NKX2.2 and ISL1, are also implicated in the late stage of EEC differentiation [3, 7].

Because there are similar pathways of differentiation between EECs and β-cells, it is not surprising that intestinal crypt cells or enteroendocrine progenitor cells have been coaxed into insulin-expressing cells for many years [8–13]. In particular, Dr. Accili’s group [14] reported that genetic inactivation of FOXO1 transcription factor in mice resulted in the expansion of NGN3-positive enteroendocrine progenitor cells, and appearance of functional insulin-producing cells that expressed all markers of mature β-cells, secreted insulin in response to glucose and sulfonylureas, and could alleviate diabetes caused by streptozotocin. Recently, they also demonstrated that using intestinal organoids derived from human induced pluripotent stem cells, FOXO1 inhibition yielded functional insulin-producing cells [15]. Another group [16] reported that induced expression of *Pdx1*, *MafA*, and *Ngn3* promoted rapid conversion of intestinal crypt cells into endocrine cells, which coalesced into islet-like clusters below the crypt bases. These clusters expressed insulin, showed ultrastructural features of β-cells, and were able to ameliorate hyperglycemia in diabetic mice. In addition, induced expression of *Pdx1*, *MafA*, and *Ngn3* in human embryonic stem cell-derived intestinal organoids stimulated the conversion of intestinal epithelial cells into β-cell-like cells. Very recently, Ariyachet et al. [17] constructed transgenic mice to drive *Pdx1*, *MafA*, and *Ngn3* expression to the gastrointestinal enteroendocrine lineage and discovered that antral stomach EECs were converted to β-cells more effectively and fully than were intestinal EECs. They also suggested that β-cells could arise from multiple subtypes of EECs and/or their common progenitors.

Recently, we reported that enteroendocrine K cells could be reprogrammed partially to β-cells through the combined expression of *Nkx6*.*1* and *Ngn3*, and reaggregation in suspension culture [18]. Therefore, it would be interesting if other subtypes of EECs have the similar potential to give rise to β-cells. As a first step toward answering this question, we obtained five EEC subtypes, L, K, I, G and S cells, from STC-1 cells, which is a heterogenous EEC line [19], and compared gene expression profiling and insulin gene methylation between these EEC subtypes and β-cells.

## Materials and methods

### Single cell sorting by flow cytometry and establishment of EEC clones

STC-1 cells were kindly supplied by Dr. Hanahan (University of California, CA). The cells were maintained in DMEM (Invitrogen Life Technologies, Carlsbad, CA) containing 25 mM glucose and 10% fetal bovine serum (FBS), and incubated in a humidified atmosphere containing 5% CO^2^ at 37°C. The medium was changed every 2–3 days.

For single cell sorting, STC-1 cells were dispersed with 0.25% trypsin-EDTA (Invitrogen Life Technologies) after reaching 80–90% confluence, and resuspended in 1 ml DMEM with 1% FBS. After single-pass sorting by flow cytometry, each single cell was cultured in individual wells of 96-well plates. After several passages, a total of 59 clones were established.

C2C12 cells (a mouse myoblast cell line), MIN6 cells (a mouse β-cell line), mouse gut mucosa, or isolated mouse islets were used as controls. K0 cells (a K cell line), which were obtained by transfection of a GIP promoter-expressing vector into STC-1 cells and selection of transfected cells [18], were also used.

### Conventional and quantitative RT-PCR

For conventional RT-PCR, total RNA isolation, first-strand cDNA synthesis, and PCR were performed with standard procedures. The PCR products were analyzed on 2% agarose gels and visualized after staining with ethidium bromide. Expression of β-actin was used as an internal control.

For quantitative RT-PCR, total RNA was isolated from the cells using Trizol reagent (Invitrogen Life Technologies) according to the manufacturer’s instructions. Reverse transcription was carried out using the 1st Strand cDNA Synthesis kit (Promega, Madison, WI), in which 2 μg of total RNA was used as a template. One tenth of cDNA was used for quantitative PCR using EvaGreen Supermix (Takara, Shiga, Japan). The PCR protocol was: initial denaturation at 95°C for 5 min; 40 cycles of denaturation at 95°C for 20 s, annealing at 60°C for 30 s and extension at 72°C for 20 s; and melt curve from 65°C to 95°C. Expression of β-actin was used as an internal control.

The primers used for PCR are shown in S1 Table.

### Immunostaining

The cells were grown on cover glasses, and fixed in 4% paraformaldehyde for 10 min. Immunostaining was performed using the following primary antibodies: goat polyclonal anti-CCK (1:100; Santa Cruz Biotechnology, Santa Cruz, CA), goat polyclonal anti-GIP (1:100; Santa Cruz Biotechnology), goat polyclonal anti-gastrin (1:100; Santa Cruz Biotechnology), goat polyclonal anti-secretin (1:100; Santa Cruz Biotechnology) and goat polyclonal anti-GLP-1 (1:100; Abcam, Cambridge, MA). After overnight incubation with primary antibodies at 4°C, slides were incubated with FITC-conjugated anti-goat IgG (1:100) as the secondary antibody. The nuclei were stained with 4′,6′-diamidino-2-phenylindole (DAPI). The stained cells were examined with fluorescence microscope and confocal microscope.

### Hormone secretion assay

To detect hormone release, cells were seeded into 12-well culture plates (1 × 10^5^ cells/plate). When cells reached 80–90% confluence, the supernatant was removed and replaced with DMEM without FBS. After culture for 24 h, the supernatants were collected and stored at -20°C for hormone assay. CCK, gastrin, or secretin was measured using the appropriate EISKA kits from RayBiotech (Norcross, GA), and GIP and GLP-1 were simultaneously measured using a EISA kit from Merk (Darmstadt, Germany). The levels of hormones were normalized to the protein content of cells. The protein content was measured using the Bradford method.

### Microarray analysis

Microarray experiments were performed in Macrogen (Seoul, Korea) using Mouse 2.0 ST Array to analyze over 35,240 genes, including about 28,000 coding genes and about 7,000 long non-coding transcripts. Total RNA from the cells was isolated using the Qiagen RNeasy Mini kit (Qiagen GmbH, Hilden, Germany). After total RNA extraction, the RNA was treated with DNase and subjected to a second RNA purification using the Qiagen kit (Qiagen GmbH). A total of 100 ng of RNA from each sample was amplified to make cDNA using the GeneChip Whole Transcript Amplification kit according to the supplied protocol. The sense cDNA was then fragmented and biotin-labeled with terminal deoxynucleotidyl transferase using the GeneChip Whole Transcript Terminal Labeling kit. A 5500-ng sample of each cDNA was hybridized to the Affymetrix GeneChip Mouse 2.0 ST Arrays at 45°C for 16 h. Hybridized arrays were scanned using a GSC3000 Scanner.

The raw intensity data were obtained automatically from the Affymetrix data extraction protocol using the software provided by Affymetrix GeneChip Command Console Software. The data were normalized using the robust multi-average (RMA) method. The differentially expressed genes (DEGs) were analyzed based on their fold-change difference and hierarchical cluster analysis using complete linkage and Euclidean distance as a measure of similarity. All data analyses and visualization of DEGs were conducted using R 3.1.2.

These procedures were accomplished twice for each cell sample, on different days, and the mean value was used for analyses.

### Methylation assay of cytosine-guanosine dinucleotide (CpG) sites in the *Ins2* promoter

Methylation of CpG sites in the *Ins2* promoter located at -414, -182, and -171 bp relative to the transcription start site was examined, as described by Kuroda et al. [20]. Genomic DNA was isolated using the ZR genomic DNA kit (Zymo Research, Orange, CA), and treated using the EZ DNA methylation kit (Zymo Research) according to the manufacturer’s recommendations. The *Ins2* gene was amplified with the appropriate primers in a mixture containing 100 ng bisulfite-modified DNA. The following primers were used: *Ins2* bisulfite sense TTTAAGTGGGATATGGAAAGAGAGATA; *Ins2* bisulfite antisense CTACAATTTCCAAACACTTCCCTAATA; and *Ins2* antisense CTGCAGTTTCCAAACACTTCCCTGGTG. PCR was accomplished using HotStarTaq DNA polymerase (Qiagen, Valencia, CA). Each PCR fragment was extracted and sequenced with gene-specific primers. Sequencing was performed in Cosmo Genetech (Seoul, Korea). The percentage of methylation at every CpG site on *Ins* promoter was calculated as the peak height of C vs. the peak height of *C* plus the peak height of T [21].

## Results

### Establishment of L, K, I, G, and S cell clones

**Fig 1:**
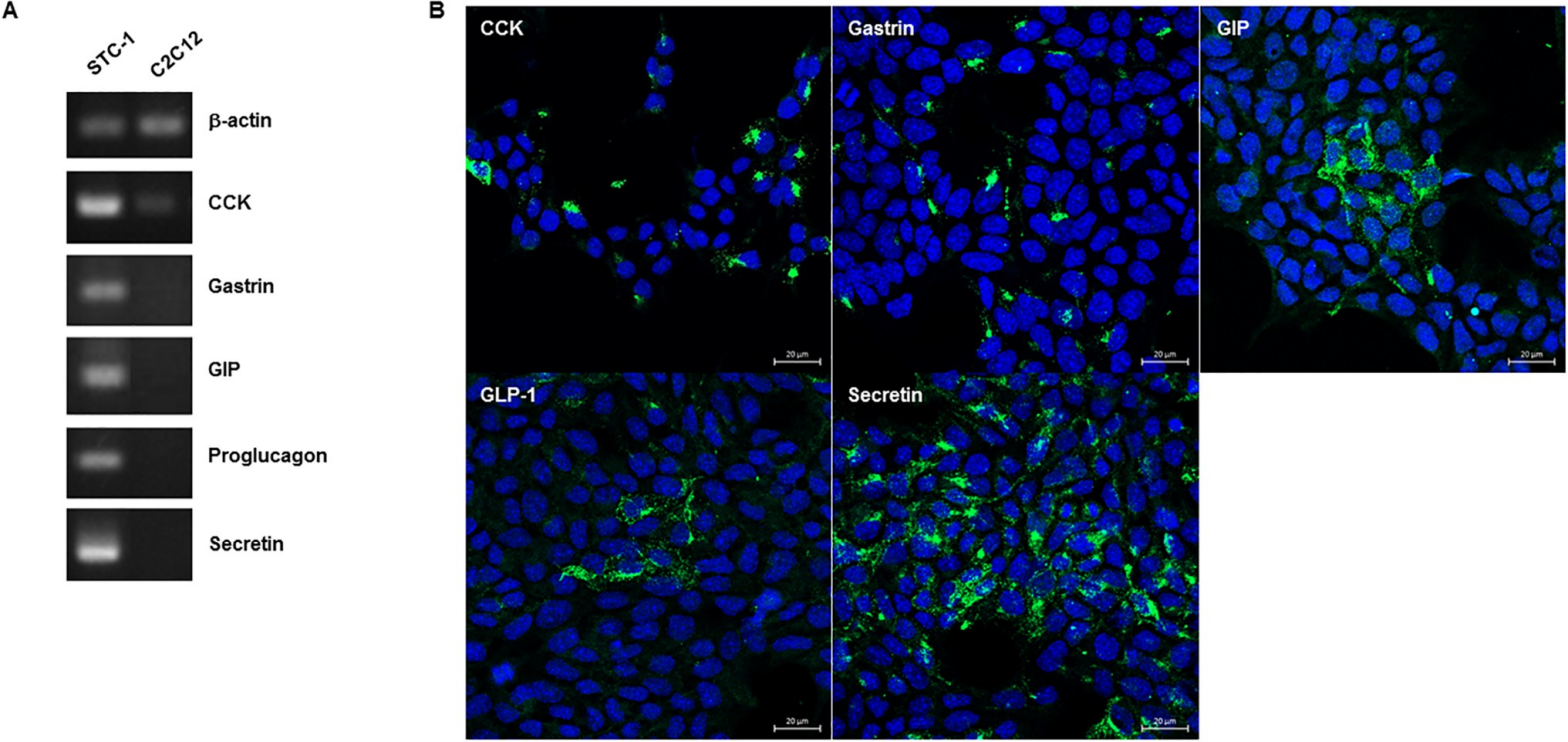
Expression of gut hormones in STC-1 cells. (A) RT-PCR showed STC-1 cells expressed mRNA transcripts of all of five gut hormones, while C2C12 cells did not. (B) Confocal images of immunostaining revealed the presence of all of five gut hormones (green). The nuclei were stained with DAPI (blue). Bar, 20μm.

**Fig 2:**
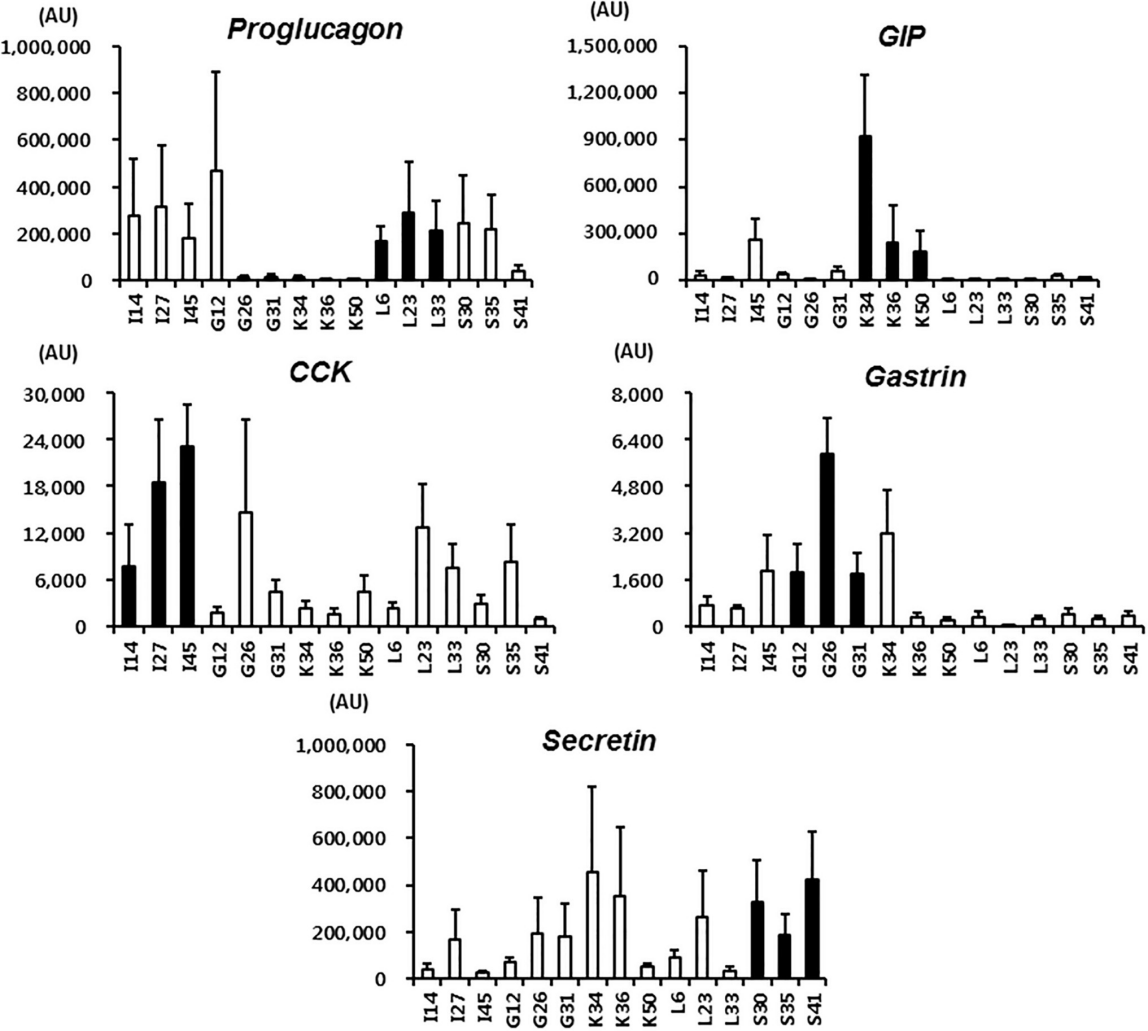
Selection of clones of EEC subtypes. Three different clones of L, K, I, G and S cells were selected using quantitative RT-PCR so that each clone of EEC subtypes had the highest expression of the corresponding hormones and the lowest expression of other hormones. Filled bars indicate three clones of L, K, I, G or S cells at passage 10, 30, and 40. Data are the mean ± the standard error of the mean. AU, artificial units.

**Fig 3:**
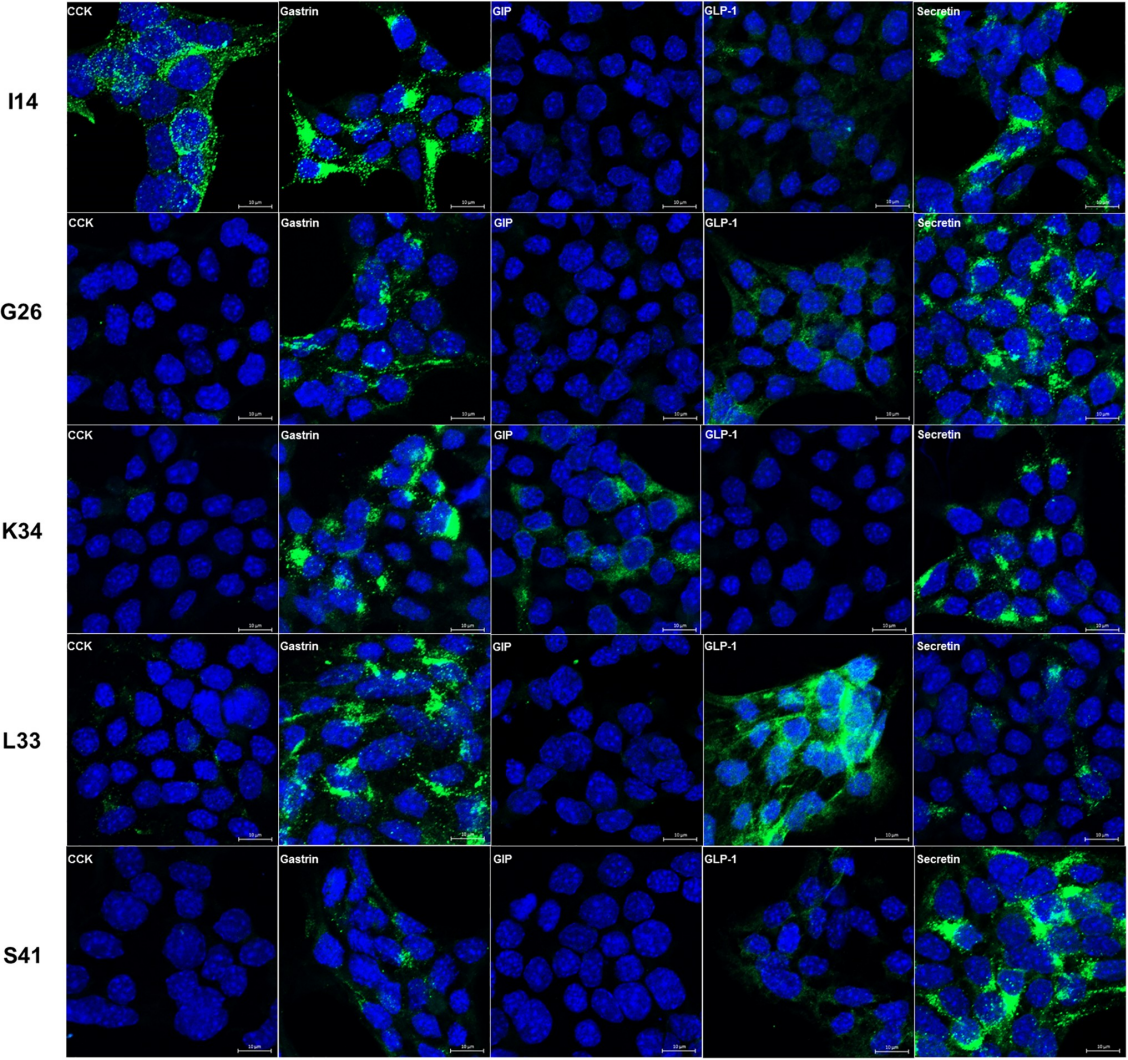
Immnostaining of the representative clones of EEC subtypes. Confocal images of immunostaining revealed the presence of gut hormones (green). The nuclei were stained with DAPI (blue). Bar, 20μm.

**Fig 4:**
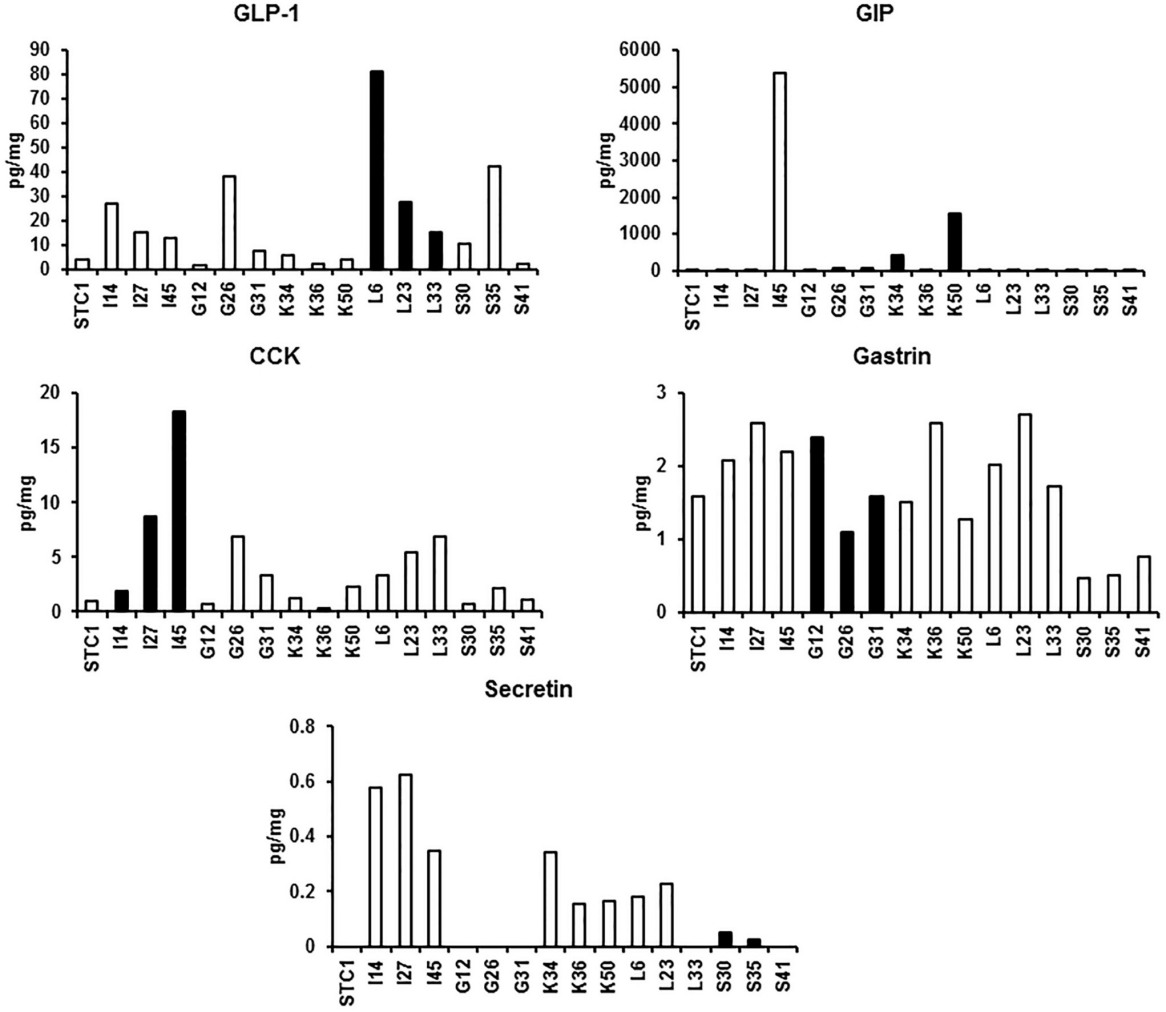
Hormone secretion of EEC subtypes. Cells were seeded into 12-well culture plates (1 × 10^5^ cells/plate). When cells reached 80–90% confluence, the supernatant was removed and replaced with DMEM without FBS. After culture for 24 h, the supernatants were collected for hormone assay. The levels of hormones were normalized to the protein content of cells. The protein content was measured using the Bradford method.

Immunostaining and RT-PCR showed that GLP-1/proglucagon, GIP, CCK, gastrin and secretin were all expressed in STC-1 cells (Fig 1A and 1B), and that secretin was the most abundant hormone. C2C12 cells, a non-endocrine cell line, did not express these hormones. Single cell culture from 100 cells of STC-1 led to the establishment of 59 clones. Since each clone coexpressed multiple hormones, we tried to select L, K, I, G, and S cell clones having the highest expression of the corresponding hormones and the lowest expression of other hormones. As a result, three different clones of L, K, I, G, and S cells were selected according to their expression of each hormone mRNA using quantitative RT-PCR (Fig 2): L6, L23 and L33 for L cells, K34, K36 and K50 for K cells, I14, I27 and I45 for I cells, G12, G26 and G31 for G cells, and S30, S35 and S41 for S cells. Immunostaining confirmed the presence of each hormone in these clones (Fig 3). As shown in RT-PCR and immunostaining (Figs 2 and 3), each EEC subtype expressed not only the corresponding hormone but also other hormones. In particular, secretin and gastrin were expressed in all EEC subtypes. Hormone secretion assay also confirmed the presence of each hormone in these clones, although secretion of secretin was very low (Fig 4).

### Global gene expression profiling

**Fig 5:**
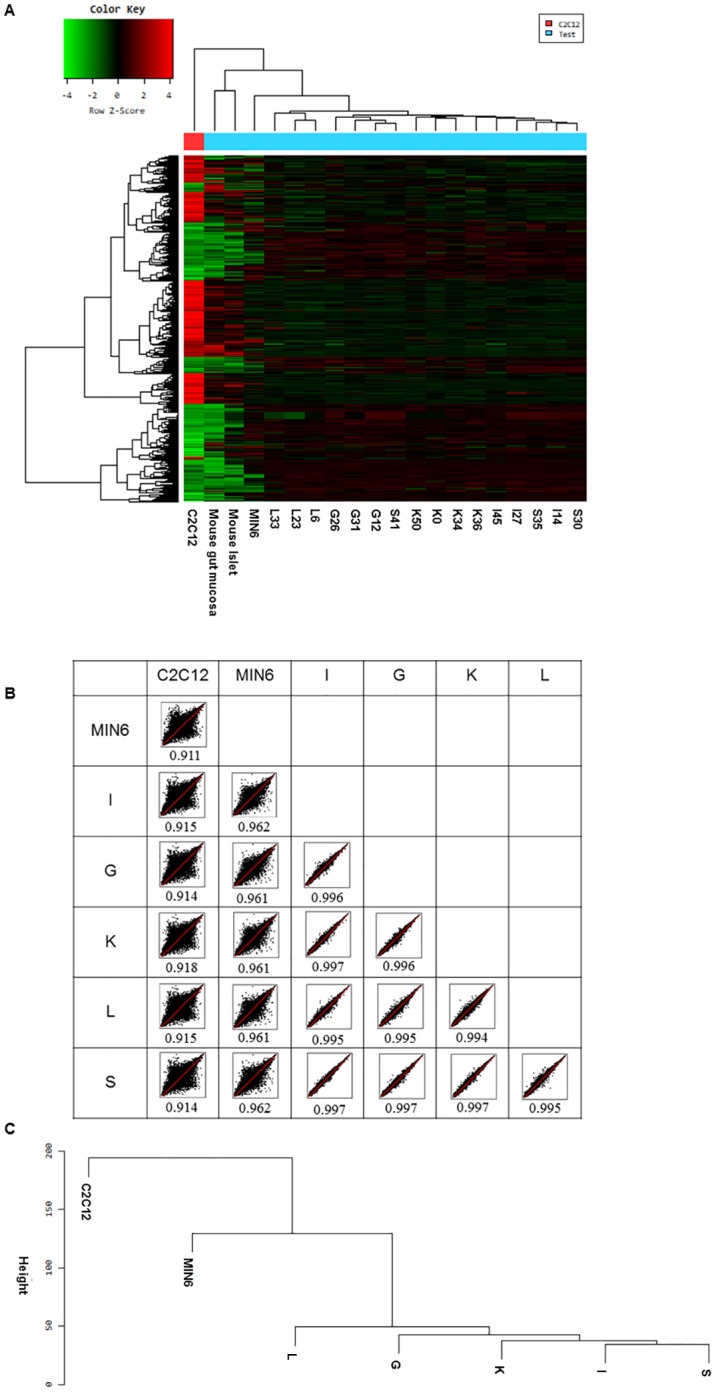
Microarray analysis of EEC subtypes. (A) Global microarray expression profiles in five EEC subtypes, MIN6 cells, mouse islets and C2C12 cells. (B) Scatter plots and associated correlation coefficients. RMA intensities of individual microarray probes were compared between different cell types and plotted on a log (10) scale. The numbers indicate the correlation coefficients. (C) Dendrogram showing the relationships between different cell types as determined by hierarchical clustering analysis. The experiments were accomplished twice for each cell sample, on different days, and the mean value was used for analyses. As for each EEC subtype, the mean values of three clones of each EEC subtype were used for analyses.

Global microarray gene expression profiles (Fig 5A) showed that a total of 1,136 DEGs were found to be greater than or equal to a threefold change of the genes expressed in all EEC subtypes compared with MIN6 cells. There were 535 up-regulated genes and 601 down-regulated genes in total DEGs that differed from MIN6 cells. By contrast, a total of 2,142 DEGs were found to be greater than or equal to a threefold change of the genes expressed in C2C12 cells compared with MIN6 cells. There were 900 up-regulated genes and 1,242 down-regulated genes in total DEGs that differed from MIN6 cells. Expression profiles of the different cell types were examined by scatter plots and associated correlation coefficients (Fig 5B). There was higher similarity between each EEC subtype and MIN6 cells (correlation coefficients = 0.961–0.962) than between C2C12 cells and MIN6 cells (correlation coefficient = 0.911). In addition, the correlation coefficients between each type of EECs were very high, 0.994–0.997, suggesting that each EEC subtype is very similar to the others.

The relationships between different cell types were further examined by hierarchical clustering analysis (Fig 5C). This confirmed that EECs are more closely related to MIN6 cells than to C2C12 cells. In addition, MIN6 cells were more closely related to L cells than to other EEC subtypes.

### Gene expression profiling for β-cell-related transcription factors

**Fig 6:**
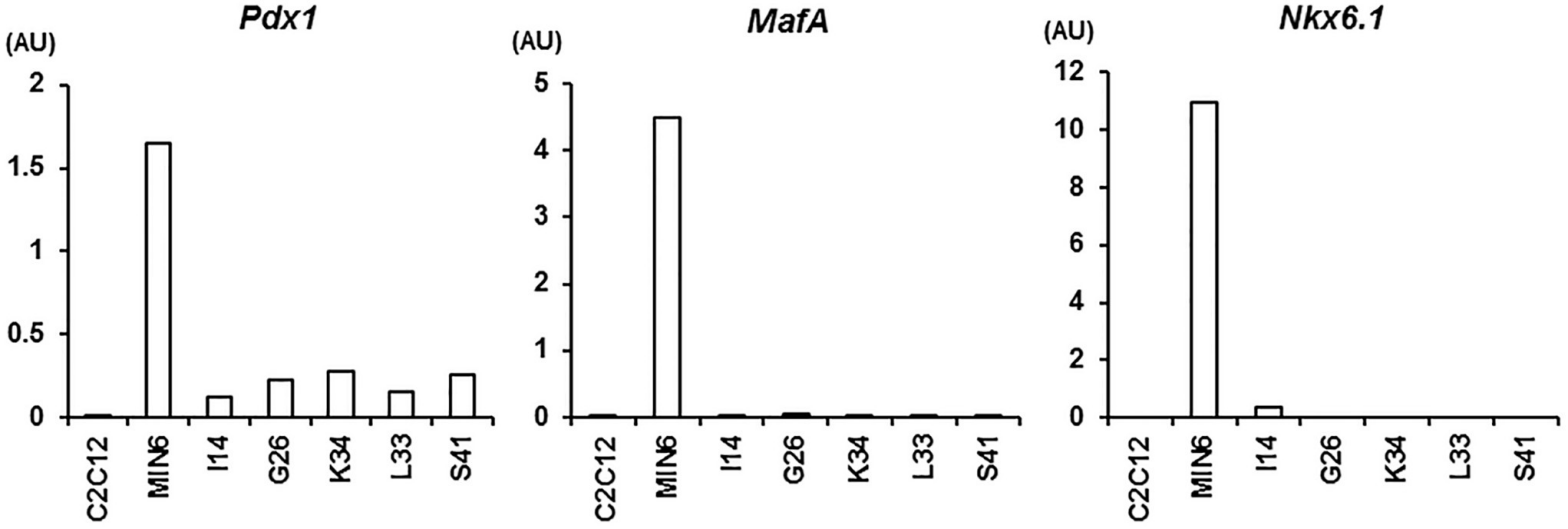
Expression of β-cell-specific transcription factors in the representative clones of EEC subtypes. Gene expressions of Pdx1, MafA and Nkx6.1 were measured in the representative clones of L, K, I, G and S cells, MIN6 cells and C2C12 cells using quantitative RT-PCR. AU, artificial units.

Next, we analyzed the microarray data for β-cell-related transcription factors, which are enriched in β-cells [22] and involved in pancreas development [23] (S2 Table). There were 14 genes with lower expression and 15 genes with higher expression in all subtypes of EECs compared with MIN6 cells as determined by a threefold or higher change (Table 1). As expected, *Pdx1*, *MafA* and *Nkx6*.*1* were among the genes with low expressions in EECs. Low expression of these three factors was confirmed by quantitative RT-PCR in the representative clone of each EEC subtype (Fig 6). In addition, we compared the microarray data for insulin secretion-related proteins in EECs and MIN6 cells, and found that most proteins except *GLUT2*, *Kir6*.*2*, and *Sur1* were expressed in both cells (Table 2).

**Table 1:** Expression levels of β-cell-related transcription factors in EECs compared with MIN6 cells.

Common names	Gene symbols	Fold change	Common names	Gene symbols	Fold change
Nkx6.1	Nkx6-1	-21.75 ± 0.11	HIF-2a	Epas1	39.77 ± 0.51
Lsr	Lsr	-17.01 ± 0.28	Foxq1	Foxq1	18.00 ± 0.81
Meis2	Meis2	-14.69 ± 0.13	Tox	Tox	11.74 ± 0.30
CHREBP	Mlxipl	-13.42 ± 0.64	Musculin	Msc	10.14 ± 0.68
Npas3	Npas3	-6.29 ± 0.44	Ets1	Ets1	8.53 ± 0.30
MafA	Mafa	-5.17 ± 0.23	Cdx2	Cdx2	6.95 ± 0.26
NR2A2	Hnf4g	-4.37 ± 0.20	Gata2	Gata2	6.06 ± 0.40
Irf6	Irf6	-4.37 ± 0.43	Fev	Fev	5.14 ± 0.68
c-Fos	Fos	-4.21 ± 0.49	Irf7	Irf7	4.61 ± 0.44
Nur77 or NGFIB	Nr4a1	-3.97 ± 0.53	MafB	Mafb	4.44 ± 0.38
IPF1	Pdx1	-3.65 ± 0.32	Tbx3	Tbx3	3.74 ± 0.52
Nupr1	Nupr1	-3.30 ± 0.85	Hoxb6	Hoxb6	3.33 ± 0.63
RORg	Rorc	-3.16 ± 0.31	Pdlim1	Pdlim1	3.09 ± 0.96
HIC5	Tgfb1i1	-3.05 ± 0.16	Sox6	Sox6	3.02 ± 0.39
			Pbx1	Pbx1	3.01 ± 0.26

Each value is expressed as the means ± standard deviation. Microarray experiments were accomplished twice for each cell sample, on different days, and the mean value was used for analyses. Data of three clones of each EEC subtypes were combined together to represent EECs.

**Table 2:** Expression levels of insulin secretion-related proteins in EECs compared with MIN6 cells.

Common names	Gene symbols	Description	Fold change
GLUT2	Slc2a2	Glucose sensing	-26.30 ± 0.36
glucokinase	Gck		2.13 ± 0.61
Kir6.2	Kcnj11		-4.07 ± 0.26
Sur1	Abcc8		-3.75 ± 0.31
Cacna1c	Cacna1c	Insulin trafficking	-1.37 ± 0.24
Cacna1d	Cacna1d		1.26 ± 0.35
Snap25	Snap25		-1.03 ± 0.60
Vamp2	Vamp2		-1.27 ± 0.43
Stx1a	Stx1a		-1.09 ± 0.39
Munc18a	Stxbp1		-1.05 ± 0.19
Munc18b	Stxbp2		-1.61 ± 0.28
Munc18c	Nsfl1c		-1.04 ± 0.16
Rab3a	Rab3a		-1.26 ± 0.21
Rab3b	Rab3b		-1.52 ± 0.35
Rab3c	Rab3c		3.81 ± 0.23
Rab3d	Rab3d		-2.94 ± 0.24
Rab27a	Rab27a		-1.67 ± 0.22
PC1/3	Pcsk1	Insulin processing	-1.51 ± 0.31
PC2	Pkd2		1.03 ± 0.35

Each value is expressed as the means ± standard deviation. Microarray experiments were accomplished twice for each cell sample, on different days, and the mean value was used for analyses. Data of three clones of each EEC subtypes were combined together to represent EECs.

**Fig 7:**
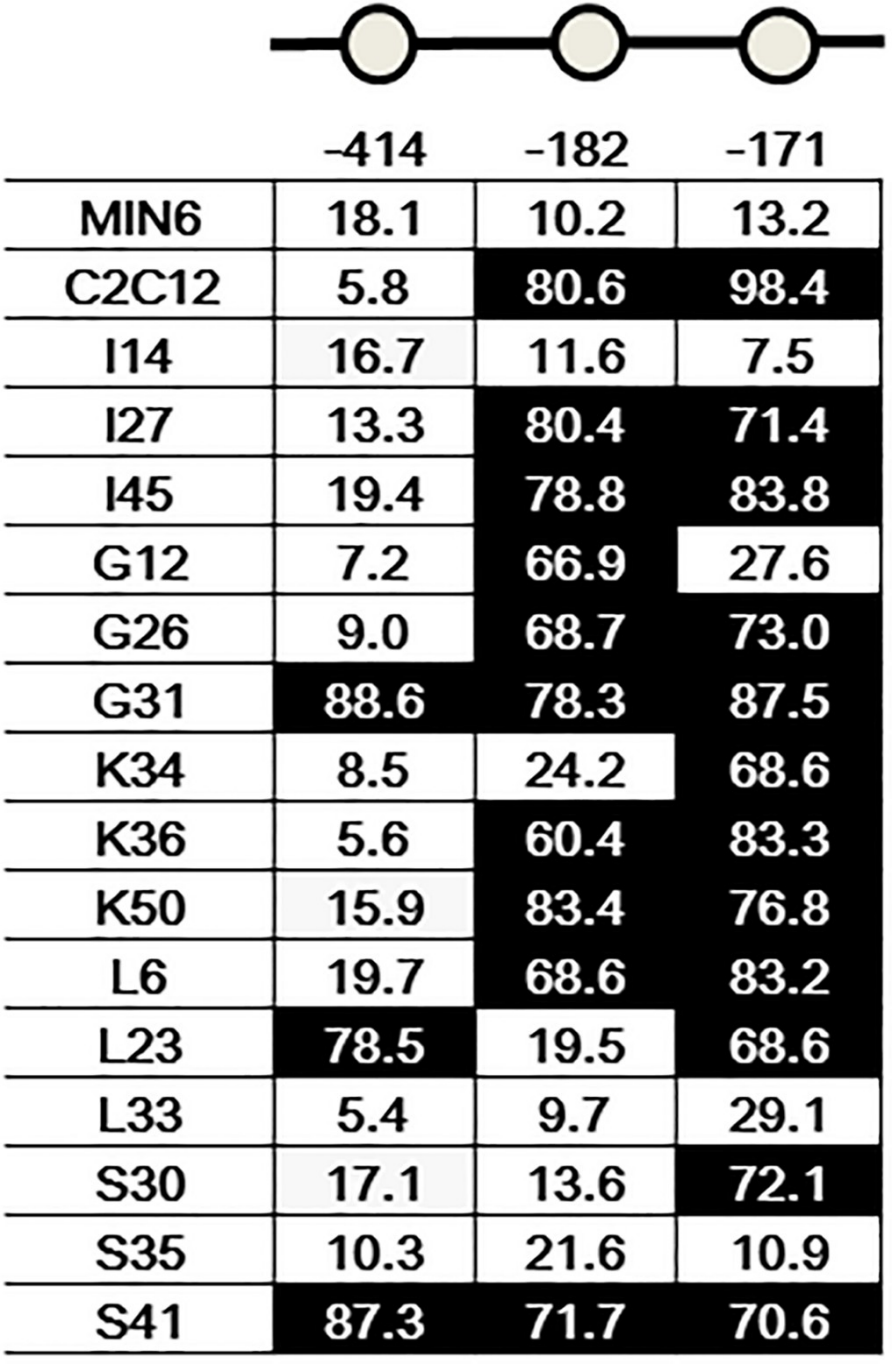
Insulin gene methylation. Methylation of CpG sites in the *Ins2* promoter located at -414, -182, and -171 bp relative to the transcription start site was examined. The number in the boxe indicates the percentage of methylation of these CpG sites. White boxes indicate <30% methylation in CpG sites.

Insulin gene methylation assay showed that three CpG sites in the *Ins2* promoter located at -414, -182, and -171 bp were fully unmethylated in MIN6 cells. The CpG site at -414 bp was mostly unmethylated in EECs, while the other CpG sites were mostly methylated in EECs. Although each EEC subtype showed variable methylation (Fig 7), one clone of I, L or S cells showed <30% methylation in all of these CpG sites. Microarray data showed that there was little expression of *Ins2* in all of EEC clones (S1 Fig).

## Discussion

In this study, we established five EEC subtype clones from STC-1 cells, and demonstrated that their global gene expression profiles are similar to those of MIN6 cells, a β-cell line, while the gene expressions of *Pdx1*, *MafA* and *Nkx6*.*1* transcription factors were very low in EECs compared with MIN6 cells.

Regenerative medicine using cell-based therapy will be a future strategy for curing patients with type 1 diabetes [2]. The similar pathways of differentiation for both EECs and β-cells make the gut an attractive target for cell-based therapy in type 1 diabetes. Several studies have demonstrated that intestinal stem cells or EECs could convert to the β-cell phenotype [8–16].

We performed a detailed investigation of the similarities between EECs and β-cells. First, we successfully established EEC subtype clones by flow cytometry-assisted single cell culture from STC-1 cells. We then selected L, K, I, G and S cell clones based on expression of their corresponding hormones. Consistent with previous studies [4–6], each EEC clone expressed multiple hormones as examined by quantitative RT-PCR and immunostaining. Thus, we chose three clones of each EEC subtype with the highest expression of its corresponding hormone and the lowest expression of other hormones.

Next, we performed microarray analysis to examine the extent to which EECs and β-cells are similar. Global microarray gene expression profiles revealed that a total of 1,136 DEGs showed a threefold or higher change in genes expressed in all EEC subtypes compared with MIN6 cells, while a total of 2,142 DEGs showed a threefold or higher change in genes expressed in C2C12 cells, a myoblast cell line, compared with MIN6 cells. Scatter plots and associated correlation coefficients using global gene expression profiles showed a high level of similarity between EECs and MIN6 cells. Hierarchical clustering analysis confirmed that EECs are closely related to MIN6 cells. Of note, this analysis also indicated that L cells were more closely related to MIN6 cells compared with other types of EECs. Each EEC subtype was found to be highly similar to each other.

To compare EECs and β-cells in greater detail, we analyzed the gene expression of β-cell-related transcription factors. Gene expressions for *Pdx1*, *MafA* and *Nkx6*.*1* transcription factors were very low in EECs compared with MIN6 cells. Quantitative RT-PCR in the representative clone of each EEC subtype confirmed these results. This finding is consistent with the fact that these transcription factors are found almost exclusively in β-cells [24, 25].

PDX1 is expressed during the early stage of β-cell development and is crucial for β-cell function. It is also abundant in STC-1 cells and strongly attributes GIP [8]. Previous reports have shown that an intestinal crypt cell line, IEC6, can be induced to express insulin after induction of PDX1 expression [9, 10].

MAFA is another crucial transcription factor in mature β-cells [26]. Induced expression of *Pdx1*, *MafA* and *Ngn3* transcription factors in pancreatic acinar cells [27] or intestinal crypt cells [16] can induce these cells to have a β-cell phenotype, suggesting an important role in β-cell reprogramming. MAFB, another MAF transcription factor, is exclusively detected in glucagon-positive cells and insulin-positive cells during mouse embryonic development; it then disappears from β-cells after birth and becomes an α-cell-specific factor [28]. The switch from MAFB to MAFA expression might be vital for functional maturation of β-cells [26]. Interestingly, our microarray data showed that EECs have less *MafA* and more *MafB* expression compared with MIN6 cells.

NKX6.1 is also expressed during the early stage of β-cell development and after birth exclusively in β-cells. Although its role in the differentiation and functions of these cells has not yet been defined, NKX 6.1 is probably necessary for β-cell maturation [29] and proliferation [30]. Gefen-Halevi et al. [31] reported that NKX 6.1 promoted the reprogramming of hepatocytes to β-cell. We also reported that K cells could be reprogrammed to β-cell through the combined expression of *Nkx6*.*1* and *Ngn3*, and reaggregation in suspension culture [18]. In addition, recent studies have reported that NKX 6.1 induced β-cell proliferation through the NR4A nuclear receptors such as NR4A1 and NR4A3, and was dependent on c-FOS transcription factor [32, 33]. Interestingly, the present study revealed that *Nr4a1* and *c-Fos* mRNA transcripts were depleted in EECs compared with MIN6 cells.

We also found that many other transcription factors were depleted or enriched in EECs compared with MIN6 cells, the exact role of these factors in the differentiation and functions of β-cells should be examined in the future.

As for insulin secretion-related proteins, their mRNA transcripts were mostly enriched in EECs, as they are in MIN6 cells.

Finally, we conducted insulin gene methylation analysis because it may play a crucial role in β-cell maturation and tissue-specific insulin gene expression [20]. This analysis revealed that three CpG sites in the *Ins2* promoter were fully unmethylated in MIN6 cells, while each EEC subtype showed variable methylation in these CpG sites. Interestingly, one clone of I, L and S cells showed full unmethylation, implying that one of these cells may be an optimal candidate for reprogramming to β-cells.

The current study has some limitations. First, MIN6 cells we used as β-cells are not 'perfect' β-cell models because they are originally from islet tumor, which means a lot of sites of genome might be demethylated. Next, there were discrepancies between hormone gene/protein expression and hormone secretion in EEC clones. Hormone expression and secretion in an endocrine cell could be discordant. In addition, hormone secretion was measured only at the basal state of the cells in the present study. More studies are needed for hormone secretion from these EECs.

In summary, we successfully established five EEC subtypes and found a very high level of similarity between them, as well as with MIN6 cells in global gene expression profiles.

Genes for insulin secretion-related proteins were mostly enriched in EECs. Similar to MIN6 cells, some EECs showed full unmethylation in three CpG sites in the insulin gene promoter. However, the gene expression of transcription factors crucial in mature β-cells such as *Pdx1*, *MafA* and *Nkx6*.*1* were remarkably low in all EEC subtypes. In conclusion, our data confirm that EECs are closely related to β-cells, suggesting their potential as targets for cell-based therapy in type 1 diabetes.

## Supporting information

S1 Table PCR primers. (DOCX)

S2 Table The list of gene symbols for β-cell-related transcription factors. (DOCX)

S1 Fig Expression levels of *Ins2* in EECs, MIN6 cells and mouse islet. (TIF)
