# Supplementary material for: Comparison of enteroendocrine cells and pancreatic β-cells using gene expression profiling and insulin gene methylation
Source: PLoS One. 2018 Oct 31;13(10):e0206401. doi: 10.1371/journal.pone.0206401 (PMC6209304; doi:10.1371/journal.pone.0206401)
Supplement: S1 Table — (DOCX) [file pone.0206401.s001.docx]

S1 Table. PCR primers

| Genes | Forward | Reverse | Size (bp) |
| --- | --- | --- | --- |
| β-actin | TGT TAC CAA CTG GGA CGA CA | GGG GTG TTG AAG GTC TCA AA | 165 |
| CCK | TGC CCT CAA CTT AGC TGG AC | AGC TTC TGC AGG GAC TAC CG | 158 |
| Gastrin | GAC CAA TGA GGA CCT GGA AC | AAA GTC CAT CCA TCC GTA GG | 166 |
| GIP | CAA TCT CAG GGA AAG GAG GA | CAT CCA GGC CAG TAG CTC TT | 111 |
| Proglucagon | CTT TGT GGC TGG ATT GCT TA | ATT TGC TGT AGT CGC TGG TG | 179 |
| Secretin | TCA GAC GGA ATG TTC ACC AG | ACT CTT CCT CCC TCA TCT GG | 366 |
| Pdx1 | AAA GCT CAC GCG TGG AAA GG | AAG TTC AAC ATC ACT GCC AGC T | 179 |
| MafA | TCA CCA CCA TCA CCA CCA | GAT GAC CTC CTC CTT GCT GA | 187 |
| Nkx6.1 | CAA ATC TTC GCC CTG GAG | CCG AGT CCT GCT TCT TCT TG | 178 |
