# Supplementary material for: Comparison of enteroendocrine cells and pancreatic β-cells using gene expression profiling and insulin gene methylation
Source: PLoS One. 2018 Oct 31;13(10):e0206401. doi: 10.1371/journal.pone.0206401 (PMC6209304; doi:10.1371/journal.pone.0206401)
Supplement: S2 Table — (DOCX) [file pone.0206401.s002.docx]

S2 Table. The list of gene symbols for β-cell-related transcription factors.

| Ablim1 | Fev | Lmo4 | Nr0b2 | Smad1 |
| --- | --- | --- | --- | --- |
| Adnp2 | Fhl2 | Lpp | Nr1d1 | Smad2 |
| Ahr | Fhl3 | Lsr | Nr1d2 | Smad3 |
| Apbb1 | Fli1 | Lztfl1 | Nr1h2 | Smad4 |
| Arid5a | Fos | Maf | Nr2c1 | Smad6 |
| Arnt2 | Fosl2 | Mafa | Nr2c2 | Smad7 |
| Arntl | Foxa2 | Mafb | Nr2f2 | Smyd3 |
| Arx | Foxa3 | Mafg | Nr2f6 | Snai2 |
| Ash1l | Foxc1 | Max | Nr3c1 | Sohlh2 |
| Atf2 | Foxj1 | Mdc1 | Nr4a1 | Sox12 |
| Atf3 | Foxj2 | Mef2a | Nr5a2 | Sox17 |
| Atf4 | Foxj3 | Mef2b | Nrbp1 | Sox4 |
| Atf6 | Foxk2 | Mef2c | Nrl | Sox5 |
| Atf7 | Foxm1 | Mef2d | Nucb2 | Sox6 |
| Atoh8 | Foxn2 | Meis1 | Nupr1 | Sox9 |
| Atrx | Foxo3 | Meis2 | Olig1 | Sp100 |
| Bach1 | Foxp1 | Mesp1 | Onecut1 | Spi1 |
| Bach2 | Foxp4 | Mga | Pax4 | Srebf2 |
| Bbx | Foxq1 | Mical1 | Pax6 | Srf |
| Brca1 | Gata2 | Mier1 | Pbx1 | Sry |
| Cdx2 | Gli3 | Mier2 | Pbx2 | Stat1 |
| Cebpa | Gmeb2 | Mier3 | Pbx3 | Stat3 |
| Cfdp1 | Grhl1 | Mlxip | Pdlim1 | Stat4 |
| Clock | Hand2 | Mlxipl | Pdlim7 | Stat5a |
| Creb1 | Hbp1 | Msc | Pdx1 | Stat5b |
| Creb3 | Hes1 | Msx1 | Per1 | Stat6 |
| Creb3l1 | Heyl | Mta1 | Phc2 | Tbx3 |
| Creb3l4 | Hhex | Mta2 | Phtf1 | Tcf12 |
| Creb5 | Hif1a | Mxd4 | Pias4 | Tcf19 |
| Crebl2 | Hnf1b | Mycbp | Pitx1 | Tcf20 |
| Crebzf | Hnf4g | Myt1l | Pknox1 | Tcf20 |
| Crem | Hopx | Ncoa2 | Pou5f1 | Tcf21 |
| Crip2 | Hoxa10 | Ncor1 | Pou6f2 | Tcf23 |
| Csrp1 | Hoxb6 | Neurod1 | Ppara | Tcf3 |
| Ctdsp1 | Id1 | Neurog3 | Ppard | Tcf4 |
| Ctnnb1 | Id2 | Nfat5 | Prickle2 | Tcf7l1 |
| Dach2 | Id4 | Nfatc2 | Prox1 | Tcf7l2 |
| Ddit3 | Ift57 | Nfatc3 | Prrx1 | Tead2 |
| Deaf1 | Ikzf4 | Nfatc4 | Ptf1a | Tes |
| Dek | Insm1 | Nfe2l1 | Rara | Tfcp2 |
| Dennd4a | Irf2 | Nfib | Rarg | Tfe3 |
| Dmtf1 | Irf5 | Nfic | Rcor1 | Tfeb |
| E2f1 | Irf6 | Nfix | Rela | Tgfb1i1 |
| E2f4 | Irf7 | Nfkb1 | Relb | Tgif1 |
| E2f5 | Irx2 | Nfkb2 | Rere | Tgif2 |
| Ehf | Irx3 | Nfkbia | Rfx2 | Tgif2lx2//Tgif2lx1 |
| Elf1 | Isl1 | Nfrkb | Rfx6 | Thra |
| Elf3 | Jun | Nfx1 | Rnf24 | Thrb |
| Elk1 | Jund | Nipbl | Rnf4 | Tmf1 |
| Elk4 | Klf5 | Nkx1-2 | Rorc | Tox |
| Epas1 | Lasp1 | Nkx2-2 | Runx1 | Usf2 |
| Erf | Ldb1 | Nkx3-2 | Runx1t1 | Whsc1 |
| Ets1 | Lima1 | Nkx6-1 | Rxra | Xbp1 |
| Etv1 | Lims1 | Nkx6-2 | Rxrb | Ybx1 |
| Etv6 | Litaf | Npas2 | Satb1 | Zbtb7a |
| Eya3 | Lmcd1 | Npas3 | Sbno1 | Zhx2 |
| Ezh1 | Lmo1 | Nr0b1 | Six2 | Zyx |
