# Supplementary figures and images for: Comparison of enteroendocrine cells and pancreatic β-cells using gene expression profiling and insulin gene methylation
Source: PLoS One. 2018 Oct 31;13(10):e0206401. doi: 10.1371/journal.pone.0206401 (PMC6209304; doi:10.1371/journal.pone.0206401)

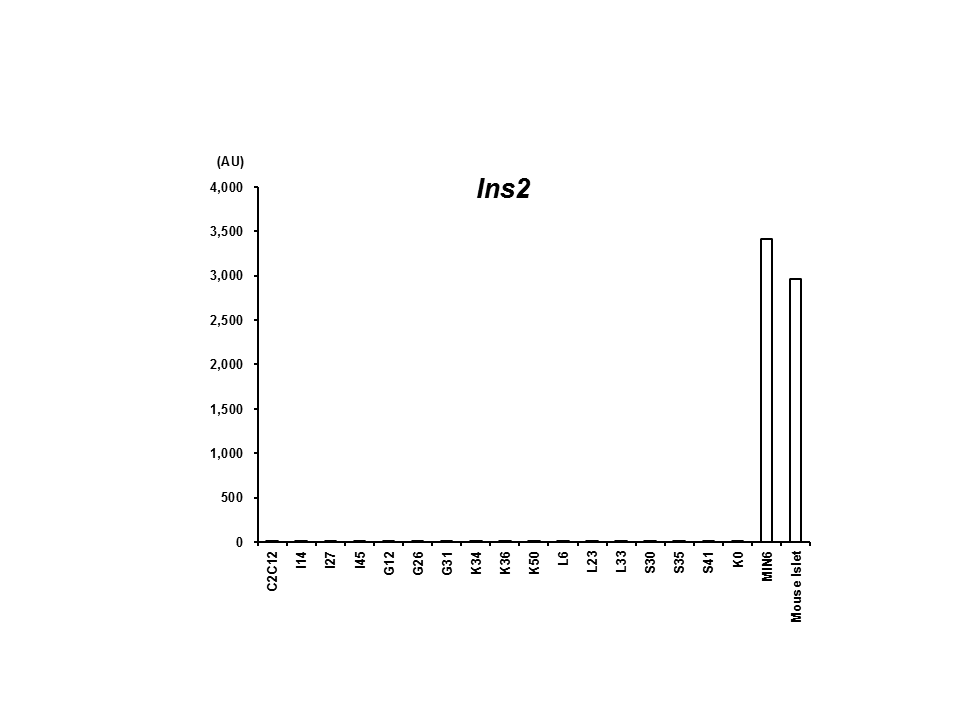

Supplement: S1 Fig — (TIF) [file pone.0206401.s003.tif]
